# Supplementary material for: Quantitative Determination of Diosmin in Tablets by Infrared and Raman Spectroscopy
Source: Molecules. 2022 Nov 27;27(23):8276. doi: 10.3390/molecules27238276 (PMC9740429; doi:10.3390/molecules27238276)
Supplement: Supplementary file 1 [file molecules-27-08276-s001.zip › molecules-2029543-supplementary.pdf]

Quantitative determination of diosmin in tablets by infrared and Raman spectroscopy

Sonia Pielorz, Magdalena Węglińska, Sylwester Mazurek, Roman Szostak

Department of Chemistry, University of Wrocław, 14F, Joliot-Curie, 50-383 Wrocław, Poland

SUPPLEMENTARY MATERIALS

Table of contents:

Fig. S1 Raman, MIR and NIR spectra of Preparation 1, diosmin hydrate, polyvinyl alcohol, croscarmellose sodium and magnesium stearate; from the top 2

Fig. S2 Experimental Raman, MIR and NIR spectra of hydrated (DSNM) and anhydrous (DSNA) diosmin 3

Fig. S3 Calculated Raman and IR spectra of hydrated (DSNM) and anhydrous (DSNA) diosmin; abscissa scale multiplied by a factor of 0.98; Gauss Lorentz profile with a half width of 8.9 cm^{-1} was used to obtain calculated spectra 3

Fig.S4 Loadings plots of PCA models obtained on the basis of Raman, DRIFTS/MIR and DRIFTS/NIR spectra of calibration samples 4

Fig. S5 Regression vectors obtained on the basis of Raman, MIR and NIR spectra of calibration samples 4

Tab. S1 Basic data on the analyzed preparations 5

Tab. S2 Composition of calibration samples [mf] 6

Tab. S3 API content in the studied preparations [mf] 8

Tab. S4 Band assignment in the Raman and MIR spectra of diosmin 8

Tab. S5 Parameters of PLS models for preparations 2-8 9

Tab. S6 Diosmin recovery [%] (n=10) 11

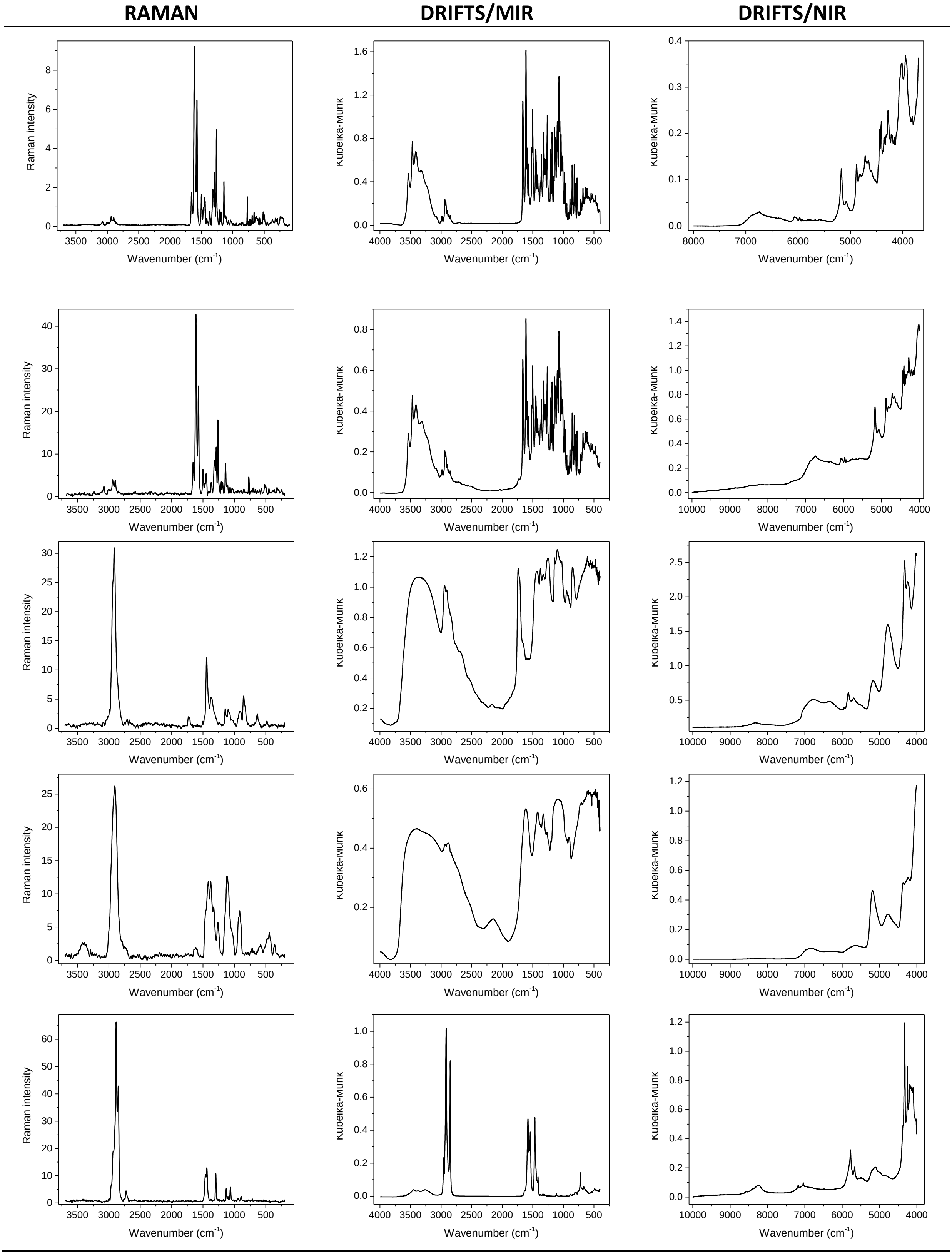

Fig. S1 Raman, MIR and NIR spectra of Preparation 1, diosmin hydrate, polyvinyl alcohol, croscarmellose sodium and magnesium stearate; from the top

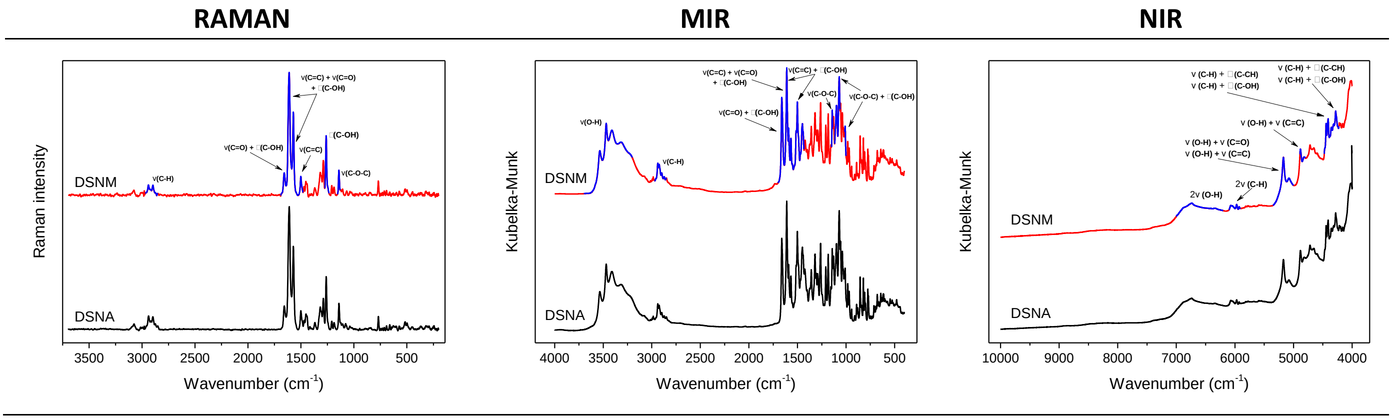

Fig. S2 Experimental Raman, MIR and NIR spectra of hydrated (DSNM) and anhydrous (DSNA) diosmin

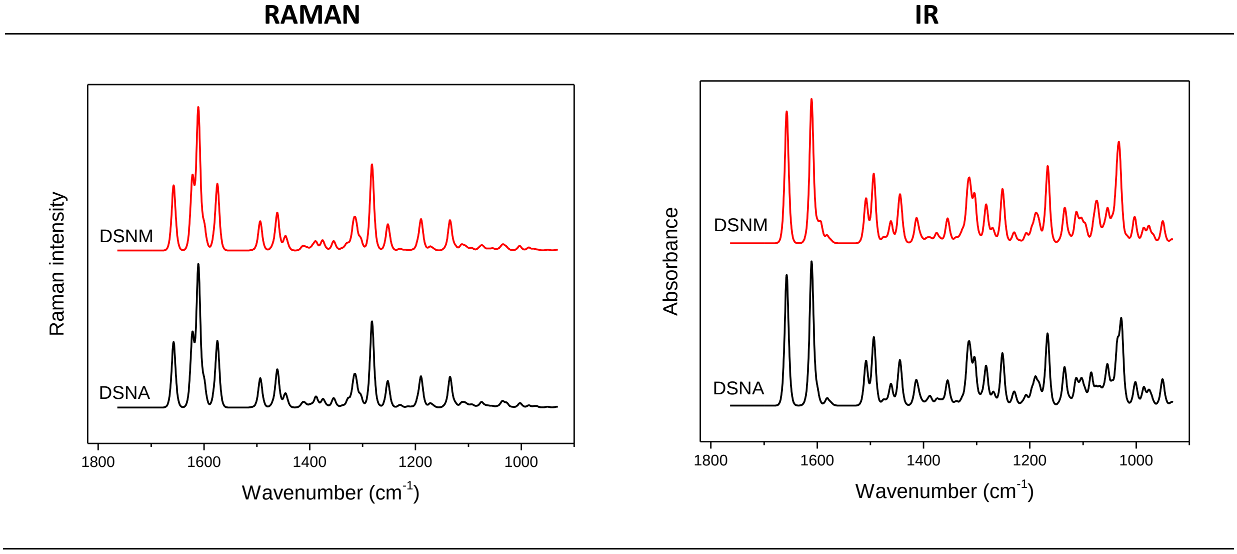

Fig. S3 Calculated Raman and IR spectra of hydrated (DSNM) and anhydrous (DSNA) diosmin; abscissa scale multiplied by a factor of 0.98; Gauss Lorentz profile with a half width of 8.9 cm^{-1} was used to obtain calculated spectra

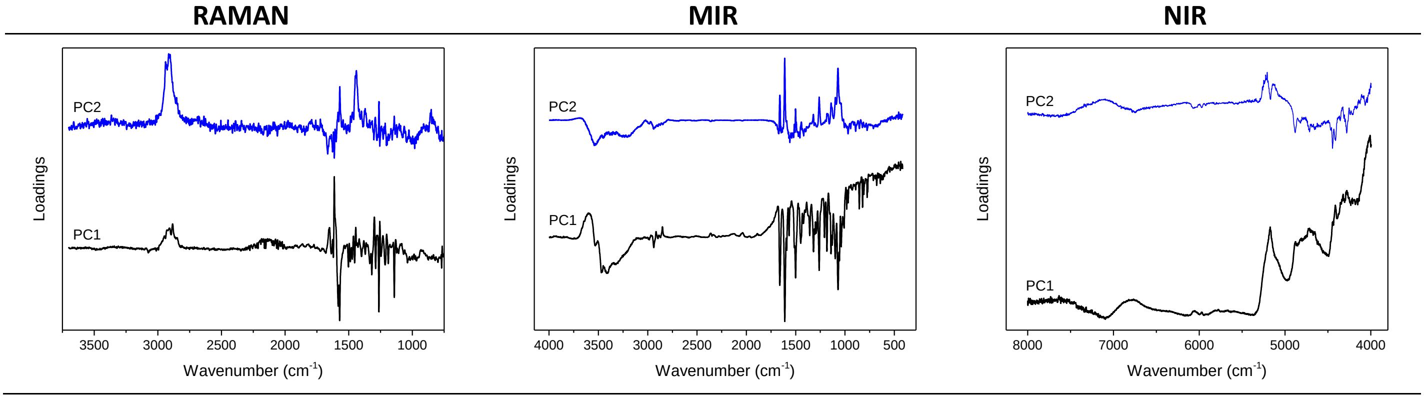

Fig.S4 Loadings plots of PCA models obtained on the basis of Raman, DRIFTS/MIR and DRIFTS/NIR spectra of calibration samples

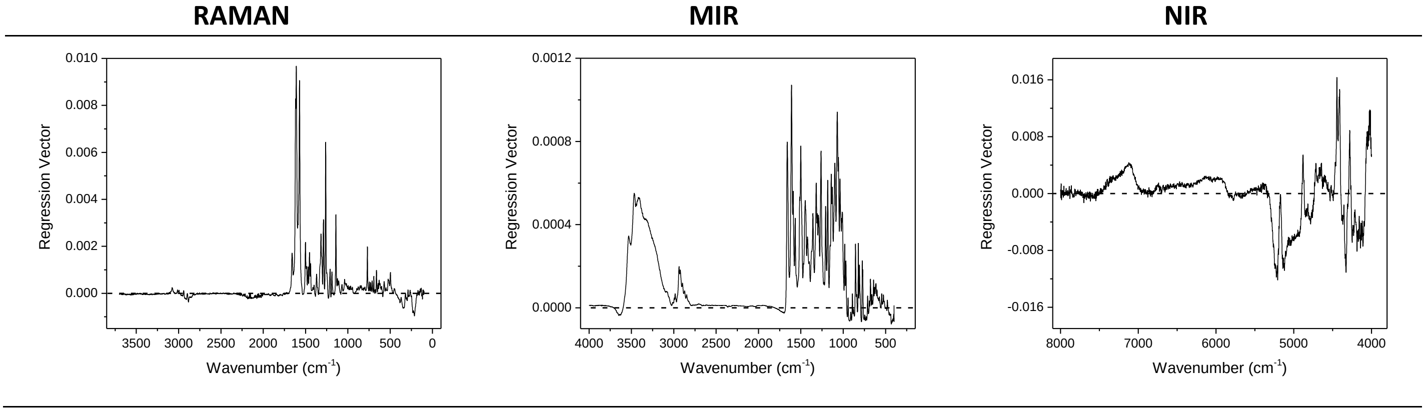

Fig. S5 Regression vectors obtained on the basis of Raman, MIR and NIR spectra of calibration samples

Tab. S1 Basic data on the analyzed preparations

Preparation	Composition of the tablet core	Declared active substance content	
		[mg/tablet]	[mf]
**Preparation 1**	Polyvinyl alcohol, croscarmellose sodium, magnesium stearate	1000	0.894
**Preparation 2**	Microcrystalline cellulose, lactose monohydrate, croscarmellose sodium, colloidal silica, magnesium stearate	1000	0.686
**Preparation 3**	Polyvinyl alcohol, croscarmellose sodium, magnesium stearate, talcum, colloidal anhydrous silica	1000	0.853
**Preparation 4**	Microcrystalline cellulose, talc, colloidal anhydrous silica, stearic acid, povidone, sodium dodecyl sulfate	500	0.653
**Preparation 5**	Microcrystalline cellulose, sodium carboxymethyl starch, magnesium stearate	500	0.783
**Preparation 6**	Polyvinyl alkohol, croscarmellose sodium, talc, colloidal anhydrous silica, magnesium stearate	1000	0.868
**Preparation 7**	Corn starch, microcrystalline cellulose, magnesium stearate, povidone	600	0.834
**Preparation 8**	Sodium carboxymethyl starch, microcrystalline cellulose, gelatin, talc, magnesium stearate	500	0.696

mf- mass fraction

Tab. S2 Composition of calibration samples [mf]

No	Diosmin	Magnesium stearate	Polyvinyl alcohol	Croscarmellose sodium
1	0.841	0.019	0.070	0.071
2	0.844	0.018	0.082	0.056
3	0.848	0.012	0.077	0.064
4	0.852	0.032	0.053	0.063
5	0.858	0.035	0.044	0.063
6	0.862	0.017	0.066	0.055
7	0.864	0.029	0.072	0.035
8	0.865	0.027	0.082	0.026
9	0.867	0.025	0.061	0.047
10	0.868	0.016	0.065	0.050
11	0.873	0.020	0.080	0.028
12	0.875	0.018	0.078	0.029
13	0.878	0.031	0.075	0.017
14	0.880	0.019	0.079	0.022
15	0.880	0.018	0.069	0.033
16	0.884	0.017	0.075	0.024
17	0.887	0.027	0.050	0.036
18	0.890	0.011	0.078	0.020
19	0.894	0.006	0.028	0.073
20	0.894	0.022	0.046	0.038
21	0.898	0.026	0.016	0.060
22	0.903	0.010	0.064	0.022
23	0.904	0.019	0.060	0.017
24	0.908	0.010	0.044	0.039
25	0.914	0.012	0.032	0.042
26	0.919	0.025	0.021	0.035
27	0.926	0.028	0.017	0.028
28	0.668	0.033	0.124	0.175
29	0.640	0.028	0.195	0.138
30	0.720	0.041	0.188	0.051
31	0.695	0.025	0.093	0.187
32	0.662	0.036	0.103	0.199
33	0.684	0.023	0.091	0.202
34	0.717	0.027	0.065	0.190
35	0.733	0.045	0.141	0.080
36	0.720	0.013	0.164	0.103
37	0.614	0.054	0.160	0.172
38	0.718	0.029	0.176	0.077
39	0.734	0.042	0.078	0.147
40	0.790	0.023	0.159	0.028
41	0.603	0.034	0.222	0.141
42	0.732	0.036	0.082	0.150
43	0.749	0.042	0.125	0.084
44	0.610	0.019	0.186	0.184
45	0.784	0.012	0.174	0.030
46	0.710	0.045	0.167	0.078
47	0.728	0.009	0.124	0.140
48	0.836	0.047	0.059	0.058

49	0.725	0.038	0.050	0.187
50	0.749	0.013	0.050	0.188
51	0.740	0.051	0.085	0.123
52	0.704	0.038	0.099	0.158
53	0.774	0.034	0.096	0.096
54	0.727	0.005	0.129	0.139
55	0.763	0.047	0.036	0.154
56	0.712	0.050	0.062	0.176
57	0.732	0.038	0.032	0.198
58	0.608	0.028	0.196	0.168
59	0.830	0.038	0.097	0.034
60	0.702	0.051	0.179	0.068
61	0.716	0.028	0.174	0.082
62	0.763	0.050	0.144	0.043
63	0.732	0.021	0.177	0.070
64	0.652	0.029	0.172	0.148
65	0.704	0.040	0.183	0.073
66	0.737	0.025	0.081	0.158
67	0.791	0.020	0.047	0.142
68	0.708	0.042	0.158	0.093
69	0.713	0.007	0.159	0.122
70	0.734	0.016	0.095	0.156
71	0.747	0.039	0.098	0.116
72	0.754	0.022	0.080	0.144
73	0.762	0.039	0.109	0.091
74	0.774	0.011	0.124	0.091
75	0.786	0.044	0.085	0.086
76	0.791	0.032	0.055	0.122
77	0.828	0.017	0.091	0.064
78	0.839	0.016	0.082	0.063
79	0.794	0.010	0.047	0.149
80	0.802	0.023	0.093	0.082
81	0.810	0.041	0.071	0.077
82	0.825	0.013	0.098	0.064
83	0.836	0.014	0.064	0.086

mf- mass fraction

Tab. S3 API content in the studied preparations [mf]

Preparation	Diosmin		Hesperidin
	Declared	Determined by HPLC	
Preparation 1	0.894	0.902 ± 0.002	0.032 ± 0.001
Preparation 2	0.686	0.715 ± 0.003	0.024 ± 0.000
Preparation 3	0.853	0.918 ± 0.004	0.021 ± 0.001
Preparation 4	0.653	0.662 ±0.001	0.035 ± 0.000
Preparation 5	0.783	0.791 ± 0.002	0.037 ± 0.001
Preparation 6	0.868	0.907 ± 0.002	0.021 ± 0.000
Preparation 7	0.834	0.811 ± 0.003	0.014 ± 0.001
Preparation 8	0.696	0.726 ± 0.001	0.030 ± 0.000

Tab. S4 Band assignment in the Raman and MIR spectra of diosmin

Band position [cm^{-1}]		
**RAMAN**	**MIR**	**Assignment**
-	3535	ν(O-H)
-	3469	ν(O-H)
-	3411	ν(O-H)
-	3318	ν(O-H)
2940	2939	ν(CH_3)
2920	2924	ν(C-H)
2897	2897	ν(C-H)
2851	2849	ν(C-H)
1657	1661	ν(C=O), □(C-OH)
1611	1611	ν(C=C), □(C-OH)
-	1594	□(H-OH)$_{water}$
1572	1567	ν(C=C), □(C-OH)
-	1514	ν(C=C), □(CC-H)
1501	1501	ν(C=C), □(CC-H), □(C-OH)
1470	1470	□(CH_3)
1453	1449	□(CH_3)
1443	1442	□(CH_3)
1369	-	□(CC-H), □(C-OH)
-	1356	□(CC-H), □(C-OH)
1318	1320	□(CC-H)
1309	1306	□(CC-H)
1289	1293	□(C-OH), □(CC-H)
1262	1262	□(C-OH), □(CC-H)
1209	1209	□(C-OH), □(CC-H), □(CH_3)
1190	1184	□(C-OH), □(CC-H)
1142	1142	ν (C-O-C), □(C-OH)
-	1098	ν(C-O-C), ν(C-OH), □(C-OH)
1079	1074	ν(C-O-C), ν(C-OH), □(C-OH)
-	1004	ν(C-C), ν(C-OH), ν(C-O)
769	770	□(C-OH)

Tab. S5 Parameters of PLS models for preparations 2-8

		Preparation 2	
**PARAMETER**	**RAMAN**	**DRIFTS / MIR**	**DRIFTS / NIR**
R_{cal}	0.9856	0.9738	0.9840
R_{test}	0.9777	0.9748	0.9723
R_{cv}	0.9543	0.9554	0.9532
$RSEP_{cal}$	1.76	2.07	1.77
$RSEP_{test}$	2.04	2.11	2.03
Number of PLS factors	5	5	6
Wavenumber range [cm^{-1}]	680-790<br>1051-1640<br>3037-3127	1046-1119<br>1167-1196<br>1547-1639<br>2636-3118	3802-4778
Normalization	None	SNV	None

		Preparation 3	
**PARAMETER**	**RAMAN**	**DRIFTS / MIR**	**DRIFTS / NIR**
R_{cal}	0.9827	0.9680	0.9912
R_{test}	0.9767	0.9627	0.9883
R_{cv}	0.9442	0.9544	0.9535
$RSEP_{cal}$	1.85	2.37	1.22
$RSEP_{test}$	2.16	2.44	1.29
Number of PLS factors	5	5	6
Wavenumber range [cm^{-1}]	959-1640<br>3035-3122	1490-1635<br>2636-3094	4141-4919
Normalization	SNV	MSC	MSC

		Preparation 4	
**PARAMETER**	**RAMAN**	**DRIFTS / MIR**	**DRIFTS / NIR**
R_{cal}	0.9955	0.9750	0.9905
R_{test}	0.9935	0.9726	0.9837
R_{cv}	0.9507	0.9612	0.9618
$RSEP_{cal}$	0.98	2.09	1.32
$RSEP_{test}$	1.12	2.00	1.55
Number of PLS factors	6	5	7
Wavenumber range [cm^{-1}]	486-752<br>1054-1528<br>2636-3150	503-533<br>868-928<br>1168-1195<br>1546-1634<br>2563-3027	3811-3936<br>4379-4761<br>6153-6683
Normalization	SNV	MSC	SNV

		Preparation 5	
**PARAMETER**	**RAMAN**	**DRIFTS / MIR**	**DRIFTS / NIR**
R_{cal}	0.9913	0.9782	0.9844
R_{test}	0.9756	0.9666	0.9783
R_{cv}	0.9627	0.9610	0.9616
$RSEP_{cal}$	1.34	1.97	1.73
$RSEP_{test}$	1.39	2.21	1.82
Number of PLS factors	5	6	6
Wavenumber range [cm^{-1}]	486-910	1232-1390	3729-3910
	1221-1529	1390-1632	4232-4430
	3042-3125	2394-3471	5409-6624
Normalization	SNV	SNV	SNV

		Preparation 6	
**PARAMETER**	**RAMAN**	**DRIFTS / MIR**	**DRIFTS / NIR**
R_{cal}	0.9842	0.9919	0.9754
R_{test}	0.9719	0.9857	0.9767
R_{cv}	0.9533	0.9545	0.9552
$RSEP_{cal}$	1.76	1.17	2.1
$RSEP_{test}$	2.33	1.57	2.0
Number of PLS factors	5	6	6
Wavenumber range [cm^{-1}]	959-1633	951-1046	3741-3911
	3035-3121	1085-1117	4141-4436
		1547-1631	4533-5117
		3026-3698	5756-6145
Normalization	MSC	MSC	SNV

		Preparation 7	
**PARAMETER**	**RAMAN**	**DRIFTS / MIR**	**DRIFTS / NIR**
R_{cal}	0.9947	0.9683	0.9917
R_{test}	0.9881	0.9645	0.9806
R_{cv}	0.9582	0.9539	0.9626
$RSEP_{cal}$	1.05	2.36	1.23
$RSEP_{test}$	1.39	2.38	1.68
Number of PLS factors	6	5	7
Wavenumber range [cm^{-1}]	486-910	1494-1635	3811-3967
	1275-1479	2636-3094	4379-4762
	3042-3121		6153-6683
Normalization	SNV	MSC	SNV

		Preparation 8	
**PARAMETER**	**RAMAN**	**DRIFTS / MIR**	**DRIFTS / NIR**
R_{cal}	0.9913	0.9785	0.9842
R_{test}	0.9756	0.9727	0.9761
R_{cv}	0.9627	0.9576	0.9630
$RSEP_{cal}$	1.34	1.96	1.75
$RSEP_{test}$	1.39	2.01	1.79
Number of PLS factors	5	6	6
Wavenumber range [cm^{-1}]	486-910	1232-1390	3729-3910
	1221-1529	1390-1632	4232-4463
	3042-3125	2394-3726	5409-6624
Normalization	SNV	SNV	SNV

R- correlation coefficient , R_{CV}- correlation coefficient of crossvalidation, cal - calibration samples set, test – test samples set
SNV- standard normal variate, MSC- multiplicative scatter correction

Tab. S6 Diosmin recovery [%] (n=10)

Preparation	RAMAN	DRIFTS / MIR	DRIFTS / NIR
Preparation 1	99.75 $\pm$ 1.88	100.35 $\pm$ 1.42	99.91 $\pm$ 3.20
Preparation 2	99.45 $\pm$ 1.53	100.37 $\pm$ 1.04	100.49 $\pm$ 1.89
Preparation 3	99.71 $\pm$ 0.95	99.80 $\pm$ 1.64	100.02 $\pm$ 0.58
Preparation 4	99.95 $\pm$ 1.17	100.55 $\pm$ 0.87	100.25 $\pm$ 1.09
Preparation 5	100.36 $\pm$ 1.95	100.19 $\pm$ 2.83	100.41 $\pm$ 1.25
Preparation 6	99.79 $\pm$ 0.61	99.92 $\pm$ 3.40	99.84 $\pm$ 0.82
Preparation 7	100.27 $\pm$ 2.50	100.03 $\pm$ 2.69	100.00 $\pm$ 1.56
Preparation 8	100.26 $\pm$ 1.67	100.23 $\pm$ 1.57	99.96 $\pm$ 1.60
